# P073 Double vision and a difficult diagnosis

**DOI:** 10.1093/rap/rkag113.103

**Published:** 2026-11-03

**Authors:** Sajeel Ahmed, Kenzie Maginnis, Jessica Weightman

**Affiliations:** South Tyneside and Sunderland NHS Foundation Trust, Sunderland, United Kingdom; South Tyneside and Sunderland NHS Foundation Trust, Sunderland, United Kingdom; South Tyneside and Sunderland NHS Foundation Trust, Sunderland, United Kingdom

## Abstract

**Introduction:**

Giant cell arteritis (GCA) is the commonest systemic vasculitis in older adults and is classically characterised by new-onset headache, scalp tenderness, jaw claudication and visual symptoms. However, atypical presentations and negative cranial investigations can delay diagnosis and treatment. We describe a 63-year-old man presenting with sequential bilateral sixth cranial nerve palsies and headache without classical cranial symptoms. Temporal artery biopsy was negative off steroids. Extensive investigation excluded infective, malignant and autoimmune mimics before PET-CT demonstrated large-vessel vasculitis supporting a diagnosis of atypical GCA. This case highlights the diagnostic value of multimodal imaging and multidisciplinary assessment.

**Case description:**

A 63-year-old man with hypertension presented with a four-day history of severe left-sided headache associated with nausea, vomiting and peri-orbital pain in November 2024. CT brain was unremarkable and he was initially managed as presumed migraine.

He subsequently developed a left sixth cranial nerve palsy causing diplopia prompting neurological assessment. MRI brain and vascular imaging were normal, while inflammatory markers were modestly elevated (ESR 44 mm/hr, CRP 9 mg/L). Given concern for vasculitis, temporal artery biopsy was performed but showed no evidence of giant cell arteritis. Despite negative investigations, empirical treatment with intravenous methylprednisolone followed by 60 mg oral prednisolone was commenced because of high clinical suspicion. Temporal artery ultrasound performed after almost 11-days on steroids was negative.

One month later, whilst receiving prednisolone 60 mg daily, he re-presented with worsening headache and developed a contralateral sixth cranial nerve palsy. He denied jaw claudication, scalp tenderness or visual loss. Repeat inflammatory markers were only mildly elevated and the progression of symptoms despite corticosteroids prompted extensive investigation. Multiple lumbar punctures demonstrated non-specific inflammatory cerebrospinal fluid changes. Comprehensive autoimmune, infective and malignant screening, including haematological and ENT assessment, failed to identify an alternative diagnosis.

Repeated attempts to taper corticosteroids resulted in recurrent headaches, constitutional symptoms and marked rises in inflammatory markers (ESR 110, CRP 108). Severe claustrophobia delayed MRI and PET-CT imaging, necessitating hypnotherapy, while corticosteroids were rapidly tapered in the interim.

PET-CT performed off steroids, in August 2025 demonstrated faint FDG uptake involving the ascending aorta and aortic arch consistent with aortitis and large-vessel vasculitis. Following multidisciplinary review, a diagnosis of atypical large-vessel giant cell arteritis was made. Tocilizumab was commenced in October 2025, with methotrexate added five months later for ongoing steroid sparing. Diplopia resolved completely, headaches improved substantially and inflammatory markers normalised, allowing gradual corticosteroid reduction.

**Discussion:**

This case demonstrates the diagnostic challenges posed by atypical giant cell arteritis. Sequential bilateral sixth cranial nerve palsies are an uncommon manifestation of GCA and, combined with the absence of jaw claudication, scalp tenderness and visual loss, initially favoured neurological rather than vasculitic diagnoses.

A further challenge was the negative temporal artery biopsy off steroids and ultrasound after 11-days on steroids. Temporal artery biopsy is susceptible to skip lesions and therefore cannot exclude GCA in patients with a convincing clinical presentation. This case reinforces that negative cranial investigations should not provide false reassurance when suspicion remains high.

The relapsing clinical course proved equally informative. Symptom recurrence during corticosteroid tapering, accompanied by progressively rising inflammatory markers, suggested ongoing inflammatory disease despite treatment. Extensive investigation excluded infective, malignant and alternative autoimmune disorders, while repeated multidisciplinary discussion ensured the differential diagnosis continued to evolve.

PET-CT proved pivotal by demonstrating inflammation within the ascending aorta and aortic arch, supporting a diagnosis of large-vessel GCA that had not been identified by cranial investigations. This highlights the complementary role of whole-body vascular imaging in patients with suspected GCA and inconclusive temporal artery assessment, particularly when large-vessel disease is suspected.

The case also illustrates the practical challenges encountered during investigation. Severe claustrophobia delayed MRI and PET-CT, prolonging diagnostic uncertainty and emphasising the importance of adapting investigations to individual patient circumstances.

This case reinforces the point that when patients have persistent clinical suspicion for giant cell arteritis despite negative temporal artery ultrasound and biopsy, earlier PET-CT or alternative large-vessel imaging should be incorporated into the diagnostic pathway.

**Key learning points:**

• This case reinforced that giant cell arteritis should remain an important differential diagnosis in patients over 50 years presenting with new headache and cranial neuropathies, even in the absence of classical cranial symptoms. Sequential bilateral sixth cranial nerve palsies, although rare, may represent an atypical manifestation of GCA.

• Negative temporal artery ultrasound and biopsy do not exclude giant cell arteritis. Prior corticosteroid exposure, skip lesions and isolated large-vessel involvement may all reduce the diagnostic yield of cranial investigations. Clinical judgement should therefore remain central when investigations and presentation appear discordant.

• A further learning point was the importance of reassessing the diagnosis throughout the disease course. Relapsing symptoms during corticosteroid tapering, rising inflammatory markers and failure to identify an alternative diagnosis all supported ongoing inflammatory disease despite initially inconclusive investigations.

• This case also highlighted the value of multidisciplinary collaboration and multimodal imaging. PET-CT ultimately identified large-vessel inflammation and established the diagnosis after extensive neurological, infectious, malignant and autoimmune investigations had proved non-diagnostic.

• The principal learning objective from the conference would be to better understand the investigation of suspected large-vessel giant cell arteritis when temporal artery ultrasound and biopsy are negative. In particular, I would welcome discussion regarding the optimal timing of PET-CT, the influence of corticosteroid exposure on imaging sensitivity, and strategies for balancing clinical judgement with negative investigations to minimise diagnostic delay and prevent irreversible complications.

